# AGRAMP: machine learning models for predicting antimicrobial peptides against phytopathogenic bacteria

**DOI:** 10.3389/fmicb.2024.1304044

**Published:** 2024-03-07

**Authors:** Jonathan Shao, Yan Zhao, Wei Wei, Iosif I. Vaisman

**Affiliations:** ^1^Statistics and Bioinformatics Group - Northeast Area, U.S. Department of Agriculture, Agricultural Research Service, Beltsville, MD, United States; ^2^School of Systems Biology, George Mason University, Manassas, VA, United States; ^3^Molecular Plant Pathology Laboratory, U.S. Department of Agriculture, Agricultural Research Service, Beltsville, MD, United States

**Keywords:** antimicrobial peptide, AGRAMP, *Spiroplasma*, N-gram, random forest, AMP

## Abstract

**Introduction:**

Antimicrobial peptides (AMPs) are promising alternatives to traditional antibiotics for combating plant pathogenic bacteria in agriculture and the environment. However, identifying potent AMPs through laborious experimental assays is resource-intensive and time-consuming. To address these limitations, this study presents a bioinformatics approach utilizing machine learning models for predicting and selecting AMPs active against plant pathogenic bacteria.

**Methods:**

N-gram representations of peptide sequences with 3-letter and 9-letter reduced amino acid alphabets were used to capture the sequence patterns and motifs that contribute to the antimicrobial activity of AMPs. A 5-fold cross-validation technique was used to train the machine learning models and to evaluate their predictive accuracy and robustness.

**Results:**

The models were applied to predict putative AMPs encoded by intergenic regions and small open reading frames (ORFs) of the citrus genome. Approximately 7% of the 10,000-peptide dataset from the intergenic region and 7% of the 685,924-peptide dataset from the whole genome were predicted as probable AMPs. The prediction accuracy of the reported models range from 0.72 to 0.91. A subset of the predicted AMPs was selected for experimental test against *Spiroplasma citri*, the causative agent of citrus stubborn disease. The experimental results confirm the antimicrobial activity of the selected AMPs against the target bacterium, demonstrating the predictive capability of the machine learning models.

**Discussion:**

Hydrophobic amino acid residues and positively charged amino acid residues are among the key features in predicting AMPs by the Random Forest Algorithm. Aggregation propensity appears to be correlated with the effectiveness of the AMPs. The described models would contribute to the development of effective AMP-based strategies for plant disease management in agricultural and environmental settings. To facilitate broader accessibility, our model is publicly available on the AGRAMP (Agricultural Ngrams Antimicrobial Peptides) server.

## Introduction

Microbial plant diseases are a major concern worldwide, posing a significant threat to global agricultural productivity and food security. Historically, conventional approaches utilizing chemical pesticides and antibiotics have been employed to combat these diseases. Unfortunately, these methods have inherent drawbacks, including adverse impacts on the environment, collateral damage to non-target organisms, and human health. In recent years, there has been growing interest in exploring alternative approaches to plant disease management that are more sustainable and eco-friendlier. One such approach involves the use of antimicrobial peptides (AMPs).

AMPs are a diverse group of biologically active small peptides ranging from 10 to 100 amino acids in length and found in a wide variety of organisms such as plants, insects, and animals. AMPs have been studied since the 1980s following the discovery of cecropins (Steiner et al., 1981). AMPs often possess both hydrophilic and hydrophobic characteristics, making them amphipathic, which facilitates their interaction with the lipid bilayer of target cells, as the cell membrane itself is also amphipathic. This interaction between AMPs and the lipid bilayer plays a crucial role in their antimicrobial activity (Glukhov et al., 2005; Bahar and Ren, 2013). Although there is some resistance to AMPs conferred by host cell proteases, overall bacteria possess limited ability to develop resistance to AMPs, because their toxicity is usually mediated by non-specific processes as opposed to targeting a specific protein (Brender et al., 2012).

Several interaction models have been proposed to explain how AMPs interact with the cell membrane. These include (a) carpet-like, characterized by an accumulation or aggregation of AMPs; (b) toroidal pore, whose pore is characterized by polar faces of amphiphilic helices and polar headgroups of lipids which allow small molecules to pass through the pore; and (c) a barrel-stave model, whose pore is solely comprised of peptides forming a water-filled channel (Bahar and Ren, 2013; Matsuzaki, 2019). These interactions between the AMP and target cell membrane lead to a displacement of lipids in the bilayer and the consequent membrane thinning, transmembrane pore formation, altered curvature, changes in electrostatic interactions in the lipid bilayer, and localized perturbations. Membrane infiltration by the AMP might also lead to membrane rupture, and/or leakage of cellular contents through the membrane, which can be fatal to the cell (Fjell et al., 2011). Such membrane-interactive characteristics make AMPs attractive as potential alternatives to traditional antibiotics against plant pathogenic bacteria.

However, the laborious and resource-intensive nature of identifying potent AMPs through experimental assays has posed significant limitations. To address these challenges, this study utilizes a bioinformatics approach that leverages machine learning models based on N-gram representations of peptide sequences to predict and select AMPs specifically targeting plant pathogenic bacteria. While previously reported models utilize the Random Forest family of algorithms (Thomas et al., 2010; Waghu et al., 2016; Bhadra et al., 2018), this study explores N-gram representations of 2-gram and 3-gram with a 9-letter reduced alphabet and a 3-letter reduced alphabet. These representations capture the sequence patterns and motifs that contribute to the antimicrobial activity of AMPs. By training and evaluating the machine learning models using a 5-fold cross-validation technique on the training set and an independent validation set, the study assesses the predictive accuracy and robustness of the developed models in identifying AMPs.

The results indicate that the models are capable of accurately identifying AMPs against plant pathogenic bacteria, offering a more efficient and reliable alternative to traditional labor-intensive screening methods. Building upon the success of the machine learning models, the study extends its application to predicting putative AMPs encoded by intergenic regions and small open reading frames (ORFs) within the citrus genome. A laboratory test on a subset of the predicted AMPs has demonstrated strong growth inhibitory effects of these peptides against *Spiroplasma citri*, the causal agent of citrus stubborn disease, confirming the predictive capability of the machine learning models.

To enhance accessibility and facilitate broader usage, we developed a publicly available online resource called AGRAMP (Agricultural N-grams Antimicrobial Peptides).^1^ AGRAMP enables users to input FASTA-formatted sequences and obtain predictions of putative AMPs based on the trained machine-learning models. This user-friendly platform serves as a valuable tool for researchers, enabling them to identify and select potential AMPs efficiently, thereby contributing to the development of effective strategies for plant disease management in agricultural and environmental settings.

## Materials and methods

### Training and test sets—negative datasets (NOAMP)

To create the negative datasets, a multi-step process was followed. Initially, random short peptides without AMP characteristics were generated by sampling from the UniProt database,^2^ with specific search criteria applied. The UniProt database was queried in the Taxonomy search box for viridiplantae and subcellular location in the cytoplasm. Sequences with descriptions such as antibacterial, signal peptide, antiviral, antiparasitic, anticancer, spermicidal, insecticidal, secreted, and antimicrobial were excluded. This filtering yielded 125,064 protein sequences.

To further refine the dataset, these sequences were blasted against AMP sequences (targeting bacteria) in the CAMP database^3^ (Thomas et al., 2010; Waghu et al., 2014, 2016), UniProt database with matches to antimicrobial peptide, defensin, hevein, knottin, snaking and thionin, and APD database^4^ (Wang and Wang, 2004; Wang et al., 2009, 2016). Sequences with negative E-values 0.09 and lower were excluded. After this step, 81,209 protein sequences remained. Further filtering was conducted to remove miscellaneous “X” characters that can appear in protein sequences in public databases, resulting in 81,054 sequences. Since sequences in the public databases can also contain short peptides, sequences shorter than 15 bp were filtered out, resulting in 80,934 peptide sequences.

From these protein sequences, random peptides ranging from 15 to 45 amino acids in length were generated and used to create the negative dataset. The negative dataset was further curated for length to more closely match the positive training set by binning the data into bands and choosing a length randomly from the bin. This introduced some variation in the negative dataset. Sequences were then randomly selected, resulting in 1,500 sequences for the negative training set and 139 sequences for the negative test set with the length of each peptide matching closely with the positive training set. Overall, three negative datasets were created.

### Training and test sets—positive AMP datasets

A total of 2,661 AMPs that affect both gram-positive and gram-negative bacteria were obtained from the APD database.^5^ The AMPs were used to construct the training and testing sets. To reduce redundancy, the CD-hit program^6^ was employed with sequences sharing a ≥ 90 percent similarity threshold were filtered out, resulting in 2,012 sequences (Li et al., 2001; Li and Godzik, 2006). From the 2,012 peptides, those within the length range of 11–45 amino acids were retained, yielding a final set of 1,639 peptides. The 1,639 sequences were shuffled to ensure randomization. Subsequently, these 1,639 peptides were split into two sets: a training set consisting of 1,500 AMP sequences and a test set containing 139 sequences. A large percentage of peptide sequence data was kept in the training set to ensure sufficient sequences for effective training. These datasets created from APD database were used in the *in-silico* analysis for this study.

### N-grams

N-gram is a substring consisting of N characters, which is a part of a larger string, in this case the amino acid sequence of peptides. Each peptide sequence can be divided into a set of all possible overlapping N-grams. Frequencies of the N-grams with distinct compositions can be calculated and compared with their expected frequencies based on the observed frequencies of individual amino acids. The following is the equation for the N-gram likelihood used in this study (Othman et al., 2017).


$$q_{ij}=\log\frac{f_{ij}}{f_{i}f_{j}}$$

The log is used to create a distribution with positive and negative values. N-gram combinations with zero frequency were set to zero to handle the undefined value of log (0). The numerator (*f_ij_*) represents the frequency of the N-gram, while the denominator (*f_i_f_j_*) represents the frequency of the individual amino acids that make up the N-gram. Each peptide was processed through a sliding window. To reduce the compositional complexity of the peptides the natural 20-letter amino acid alphabet was replaced by smaller size alphabets (Othman et al., 2018). The study used two alphabets: one based on charge (KR ≥ B, ANCQGHILMFPSTWYV ≥ J, DE ≥ Z), and the other one based a 9-letter alphabet. In the 9-letter alphabet, the mappings are as follows: ED ≥ E, QTSNH ≥ Q, LMIVAF ≥ L, G ≥ G, W ≥ W, C ≥ C, RK ≥ R, Y ≥ Y, P ≥ P.

The number of combinations of any given N-gram is based on the formula (alphabet)^(N-gram). For example, a 3-letter 3-gram alphabet has 27 combinations, and a 9-letter 3-gram alphabet has 729 combinations. The 9-letter alphabet used in this study was developed based in part on the information from the nearest neighbor clustering of existing AMPs proposed by Veltri et al. (2018) and the basic properties of amino acids. This separates polar and non-polar and charged amino acids while giving the other amino acids their own alphabet. For example, Glycine (G) is often grouped with the hydrophobic amino acids, but the R-group is a single hydrogen. The nearest neighbor method groups Glycine (G) with Tryptophan (W), but Tryptophan’s R-group possesses a bulky ring with different properties than Glycine. And although Tyrosine (Y) and Proline have bulky side chains, each amino acid has very distinct properties, so they were separated into separate groups. Likewise, negatively charged amino acids Glutamic (E) and Aspartic Acid (D) were separated into separate groups. This proposed alphabet is intended to address the potential biases in databases as submitted peptides tend to focus on pathogens that are important to human beings.

### Bioinformatics generating putative small peptides

The citrus genome sequence data (Csinensis_154_v1.fa) was downloaded from Citrus Genome Database.^7^ The sequence data was processed to remove non-ATGC characters, especially nonsense-based NNNs. Two small peptide datasets were generated from the cleaned genome sequence. The first set of small peptides consists of open reading frames (ORFs) extracted from the intergenic regions of the citrus genome. The intergenic region extraction was performed by using the bedtools.^8^ The extracted sequences were translated using the Transeq program from the emboss suite,^9^ resulting in 1,241,730 sequences. A sampling of 10,000 ORFs was initially tested using the Random Forest Algorithm with a 2-gram program with 3-letter alphabets.

The second set of small peptides was generated from the Citrus using the MiPepid program, which is designed for micropeptide prediction (Zhu and Gribskov, 2019). This process yielded 3,232,165 sequences after selecting coding sequences. Similar to the previous step, the sequences were translated using the Transeq program from the emboss suite (see text footnote 8; Rice et al., 2000). Subsequently, the sequences were sorted for peptides that were 15–25 amino acids in length. This resulted in a final set of 685,924 short peptide sequences and they were inputted into the 2-gram and 3-gram programs using the Random Forest Algorithm. Such small peptides are often missed in traditional genome annotation practices as ORFs shorter than 150 bases are not annotated.

### Machine learning—random forest—datasets and features

The Random Forest Algorithm, implemented in Python’s Scikit-learn machine learning package (Pedregosa et al., 2011) was employed for constructing the models. The feature vectors were based on likelihoods of 3-grams with the reduced alphabets described above. Four datasets were prepared including (i) a positive set for training (positive training set), (ii) a negative set for training (negative training set), (iii) a positive set for testing (positive testing set), and (iv) a negative set for testing (negative testing set). All models were trained and evaluated using these sets. In the first part of the machine learning process, 1,500 peptides from the APD database were used as the positive test set and 1,500 peptides were used in the negative training set and the N-gram program was then tested using these sets. The positive and negative training sets were balanced evenly to minimize bias. The datasets were shuffled as input into the Random Forest Algorithm to avoid bias in the model. In addition, cross-validation (5-fold) was used for evaluation of the model where 20% of the data would be held for testing in each iteration.

The algorithm (Random Forest) classifies or recognizes a pattern on a set of data called features (N-grams likelihoods) which are characteristics or measurable properties (letters) of what is being classified (peptide). Four Random Forest models were built using a 2-gram 3 letter alphabet (9 features), (3letter^2-gram^) using reduced alphabets based on charge (model1): a 9-letter alphabet (81 features, 9 letter^2-gram^; this study; model 2); a 3-gram 3 letter alphabet (27 features, 3letter^3-gram^) using alphabets based on charge (model 3); and a 9-letter alphabet (729 features, 9 letter^3-gram^; this study; model 4). The N-gram program was also compared to a negative dataset found in the literature for comparison (Sidorczuk et al., 2022). Mathew’s correlation coefficient (MCC) and Accuracy equations were used to evaluate these models:


$$MCC=\frac{\mathrm{TPxTN}-\mathrm{FPxFN}}{\surd \left(TP+FP\right)\left(TP+FN\right)\left(TN+FP\right)\left(TN+FN\right)}$$

$$ACC=\frac{\left(TP+TN\right)}{\;\left(TP+FP+TN+FN\right)}$$

### Secondary structure prediction and amino acid properties of the AMPs

Prediction of the secondary structure of the AMPs was performed using JPred4^10^ (Drozdetskiy et al., 2015). The resulting consensus secondary structure was saved for further analysis. Since JPred4 is not effective with short peptides, each short AMP peptide was replicated and concatenated to artificially generate longer sequences. These sequences were submitted to JPred4 to get an approximation of their secondary structure. Charge density plots were graphed using EMBOSS charge^11^ (Rice et al., 2000). Pepwheels were created using EMBOSS pepwheel^12^ (Rice et al., 2000). AGGRESCAN was used to predict aggregation propensity (*in vivo* aggregation; Conchillo-Solé et al., 2007; Torrent et al., 2011; de Groot et al., 2012).

### Synthesis of putative AMPs and preparation of serially diluted solutions

The amino acid sequences of 20 putative AMPs predicted by N-gram (Supplementary Table S1) were synthesized by GeneScript (Bioch Corp, New Jersey). The synthesis was performed on the microwave-assisted PepPower™ peptide synthesis platform. The quality and purity of each synthesized peptide were examined via both mass spectrometry (MS) and high-performance liquid chromatography (HPLC) analyses. All synthesized peptides reached purity above 96%. The synthetic peptides were dissolved in nuclease-free H_2_O to make stock solutions of 5 mg/mL. The stock solutions were filtered with a 0.22 μM filter to remove any possible contaminants from the synthesis facility and were subsequently subjected to two-fold serial dilutions up to 0.1526 mg/mL.

### *Spiroplasma citri* culture

The *S. citri* strain R8A2, originally isolated from infected citrus (*Citrus sinensis*), was triply cloned, and stored in a liquid serum-free medium (LD59) at −80°C (Saglio et al., 1973; Davis et al., 2017). For this study, the strain was activated by transferring frozen culture to LD8A3 medium supplemented with 10% fetal bovine serum and incubated at 32°C until it reached the mid-log phase (approximately 10^8^ colony-forming units per mL, OD_450_ reading 0.01; Wei et al., 2022). Subsequent sub-culturing every 48–72 h was performed at 32°C, and OD measurements were performed to determine the minimum inhibitory concentration (MIC) after controlling *S. citri* growth conditions. Phenol red was used as an indicator dye to monitor culture acidity, with a color change from red to yellow indicating bacterial growth (Tully et al., 1977).

### *Spiroplasma* growth inhibition assay of predicted AMPs

*Spiroplasma citri* liquid cultures in LD8A3 medium and microtiter plates (96-well plate) were used in the laboratory assay. The antimicrobial activities of the putative AMPs were determined by using a microplate reader that tracks OD value changes at wavelength 560 nm (OD_560_) over a 48-h assay period. The previously established correlation between the OD_560_ readings and the acidity changes of the liquid *spiroplasma* culture was used as the basis for measuring the growth and multiplication of *S. citri* cells (Tully et al., 1977; Wei et al., 2022).

The master mix for the growth inhibitory assay contained 27 mL LD8A3 + 3 mL Fetal Bovine Serum, 300 μL of *S. citri* R8A2 strain mid-log phase subculture, and 1,800 μL phenol red. From this solution, 148.5 μL was removed for testing and 1.5 μL AMP (predicted) stock (100 μg/mL) was added for each peptide, respectively. The control lane contained 148.5 μL of stock culture and 1.5 μL tetracycline (TC) – 50 μg/mL, where red color is expected since *S. citri* growth would be inhibited. Another control lane contained *S. citri* inoculum without AMP (SCNOAMP), where a yellow color is expected as phenol red transitions from a red to yellow color as *S. citri* grows without inhibition.

To determine the effectiveness of the peptides against *S. citri*, the minimum inhibitory concentration (MIC) assay was conducted with different predicted AMP concentrations. Most peptides were examined at concentrations of 50 μg/mL, 25 μg/mL, and 12.5 μg/mL, while selected peptides with higher inhibition against *S. citri* were tested at concentrations of 6.25 μg/mL, 3.125 μg/mL, and 1.526 μg/mL.

The laboratory assay was repeated as stated above where each well of the assay plate contained 148.5 μL from a stock solution of 15 mL (13.5 LD8A3 plus 1.5 FB serum) Fresh LD8A3 medium, 900 μL filtered phenol red and 1.5 μL of AMP (predicted) stock or tetracycline (TC) as a positive control. LD8A3 without any peptide was used as the negative control. Each laboratory assay was performed in triplicate. All statistical analyses, including *p*-values and false discovery rate (FDR) calculations, were performed using the R statistics suite^13^ with a pair-wise t-test.

## Results

### Training and testing sets for AMP prediction models

The AMP-APD database, comprising gram-positive and gram-negative bacteria, was used to create the training set for AMP prediction models. The training set consisted of 1,500 peptides, while the testing set contained 139 peptides. In parallel, the negative dataset, NOAMP1, also contained 1,500 peptides in the training set and 139 peptides in the testing set. For training AMP and NOAMP datasets, the models utilized 2-gram and 3-gram approaches, with a reduced 9-letter alphabets grouped based on amino acid properties (this study) and a reduced alphabet based on charge. To supplement the training data, the training set EMEM, from previous studies (Sidorczuk et al., 2022) was also incorporated.

**Figure 1:**
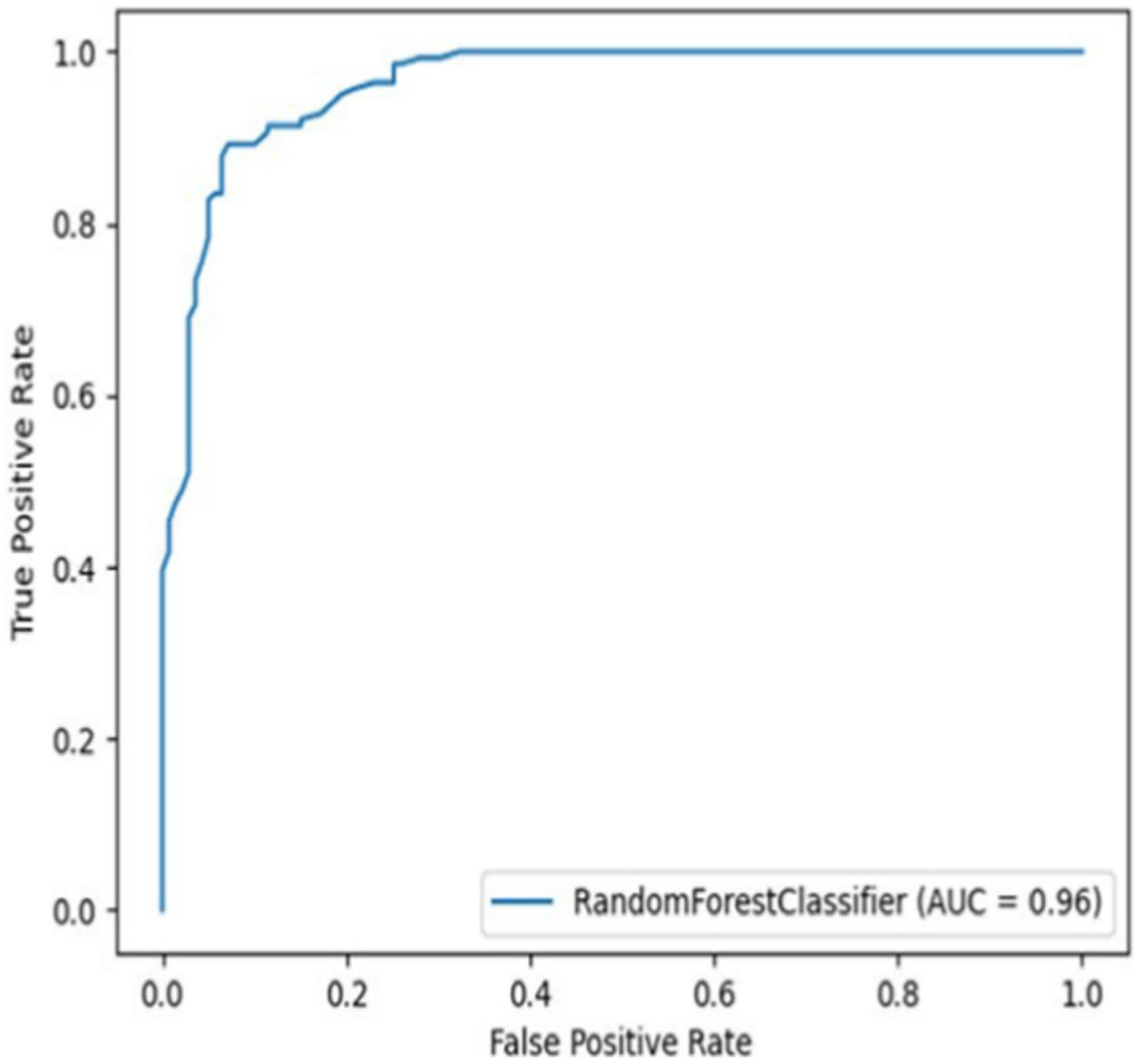
Receiver operating characteristic curve (ROC) curve for the 3-gram 9-letter alphabet model using NOAMP1 (Non-AMP dataset 1).

The models’ performance was evaluated by using 5-fold cross-validation (CR) and Mathew’s correlation coefficient (MCC), with the consistent testing datasets employed for all trials. Among these models, the 3-gram 9-letter model performed similarly but slightly better than other models, exhibiting cross-validation scores ranging from 0.88 to 0.91, and MCC values between 0.72 and 0.79 (Table 1). In addition, the receiver operating characteristic (ROC) curve showed a high true-positive rate and low false-positive rate, with an area under the curve (AUC) of 0.96 (Figure 1). The 3-gram 3-letter model based on charge demonstrated the cross-validation scores ranging from 0.77 to 0.85, and MCC values between 0.54 and 0.66 (Table 1). Similarly, the 2-gram 9-letter model performed well, displaying cross-validation scores ranging from 0.87 to 0.90, and MCC values between 0.69 and 0.82 (Table 2). The 2-gram 3-letter model based on charge exhibited the cross-validation scores ranging from 0.67 to 0.83, with MCC values between 0.57 and 0.67 (Table 2). These results indicate that the models can effectively discriminate between AMPs and NOAMPs, as demonstrated by the cross-validation scores surpassing 50%, which would be expected at random, and the AUC curve surpassing 0.5, highlighting a classifier performing better than random chance.

**Table 1:** A summary of machine learning with random forest using 3-gram with reduced alphabets.

3-gram alphabet	Datasets	Train	Test	CR1	CR2	CR3	CR4	CR5	MCC	TP	FP	FN	TN
CHARGE	NOAMP1	0.97	0.799	0.81	0.79	0.81	0.8	0.81	0.6	108	31	25	114
	NOAMP2	0.98	0.77	0.8	0.81	0.82	0.8	0.81	0.54	108	31	33	106
	NOAMP3	0.98	0.8	0.83	0.81	0.82	0.83	0.79	0.6	104	35	21	118
	EMEM	0.96	0.83	0.84	0.84	0.83	0.86	0.85	0.66	111	28	19	120
9-letter	NOAMP1	1	0.89	0.88	0.91	0.88	0.9	0.88	0.78	122	17	13	126
	NOAMP2	1	0.86	0.9	0.88	0.91	0.88	0.9	0.72	120	19	20	119
	NOAMP3	1	0.9	0.89	0.89	0.9	0.91	0.89	0.79	121	18	11	128
	EMEM	1	0.9	0.91	0.89	0.89	0.9	0.89	0.82	122	17	10	129

CR, Cross Validation; MCC, Mathew’s Correlation Coefficient; TP, True Positive; FP, False Positive; FN, False Negative; TN, True Negative.

**Table 2:** A summary of machine learning Random Forest using 2-gram with reduced alphabets.

2-gram alphabet	Datasets	Train	Test	CR1	CR2	CR3	CR4	CR5	MCC	TP	FP	FN	TN
CHARGE	NOAMP1	0.96	0.82	0.78	0.76	0.79	0.78	0.78	0.64	111	28	22	117
	NOAMP2	0.96	0.78	0.75	0.81	0.78	0.78	0.81	0.57	110	29	31	108
	NOAMP3	0.96	0.80	0.77	0.76	0.77	0.77	0.79	0.59	111	28	29	110
	EMEM	0.93	0.83	0.83	0.83	0.83	0.82	0.82	0.67	107	14	32	125
9-letter	NOAMP1	1	0.89	0.88	0.91	0.88	0.90	0.88	0.78	122	17	13	126
	NOAMP2	1	0.85	0.87	0.87	0.89	0.90	0.88	0.69	114	25	18	121
	NOAMP3	1	0.88	0.88	0.88	0.88	0.86	0.88	0.76	120	19	14	125
	EMEM	1	0.91	0.87	0.88	0.88	0.88	0.89	0.82	123	9	16	130

CR, Cross Validation; MCC, Mathew’s Correlation Coefficient; TP, True Positive; FP, False Positive; FN, False Negative; TN, True Negative.

### Machine learning for prediction of novel AMPs in citrus genome

The identification of AMPs is a complex process that involves the utilization of diverse methodologies such as Random Forest, Support Vector Machines and Deep Learning models, as documented in previous studies (Lata et al., 2007, 2010; Thomas et al., 2010; Porto et al., 2012; Veltri et al., 2018; Waghu and Idicula-Thomas, 2020; Pinacho-Castellanos et al., 2021; Wang et al., 2022). In the present study, a novel strategy was employed to enhance the identification of AMPs. Specifically, ORFs were extracted from the intergenic region, with the specific objective of identifying peptides that may not be encoded in the coding region. This approach aimed to address the possibility that certain peptides might have been overlooked in previous studies to unveil previously undiscovered peptides with distinct characteristics and potential antimicrobial properties. Furthermore, the study also involved the extraction of small peptides from the entire genome of citrus, allowing for an exploration of naturally expressed ORFs within these peptides. This comprehensive approach not only facilitated the identification of peptides but also indicated their potential for natural expression by citrus, thus implying their biological relevance and potential safety for the host organism.

By adopting the above two approaches, two datasets of small peptides were created from the intergenic region and the whole genome of citrus (details see Materials and methods). The first dataset included 10,000 randomly sampled ORFs from the intergenic region, while the second dataset consisted of 685,924 putative-predicted small peptides from the entire citrus genome. Both datasets were tested using AGRAMP (Agricultural N-gram Antimicrobial Peptides) with 3-gram and 9-letter reduced alphabet models.

### Evaluation of AGRAMP (3-gram 9-letter model) and comparison with other AMP prediction models

The peptides deduced from the intergenic region and the whole genome of citrus were analyzed by AGRAMP using a 3-gram 9-letter model. As expected, most of the unknown peptides deduced from the ORFs of the intergenic region and the putative micro-peptides from the whole genome of citrus were predicted as non-antimicrobial peptides (NOAMPs; Table 3). Approximately 7% of the 10,000-peptide dataset from the intergenic region and 7% of the 685,924-peptide dataset from the whole genome were predicted as probable AMPs by AGRAMP. AGRAMP can screen for AMP candidates in a high-throughput manner.

**Table 3:** Prediction of antimicrobial peptides (AMPs) by AGRAMP from citrus genome.

AMP probability	Intergenic region		Whole genome	
	AGRAMP	% of sample	AGRAMP	% of Sample
0.9–1	41	0.41	2,994	0.44
0.8–0.89	196	1.96	13,946	2.03
0.7–0.79	467	4.67	33,142	4.83
0.6–0.69	832	8.32	57,836	8.43
0.5–0.59	1,197	11.97	85,087	12.41
<0.5	7,267	72.67	492,919	71.86
Total	10,000	100%	685,924	100%

10,000 peptides from the intergenic region of citrus genome and 685,924 micro peptides from the whole genome of citrus were used as input.

Additionally, the performance of different AMP prediction models was evaluated using the top 10,000 probable AMPs predicted by AGRAMP and the top 10,000 NOAMPs predicted by AGRAMP. As shown in Table 4, AMP Scanner v.2 (Veltri et al., 2018) performed similarly to AGRAMP, while MACREL (Santos-Junior et al., 2020) predicted half as many AMPs, and CAMP-RF (Thomas et al., 2010; Waghu et al., 2016) predicted only 33% as probable AMPs. Compared to the other models, AGRAMP showed more positives (3,160, 7,100, and 5,459 more positives than in AMP Scanner, CAMP-RF, and MACREL, respectively). However, the predictions for the top 10,000 NOAMPs were consistent among all the models. We also compared the AGRAMP results with the predictions from AMPDiscover program using their ProtDCal-AMP_RF Random Forest model with ProtDCal descriptors^13^ (Pinacho-Castellanos et al., 2021). For this test we randomly selected 200 peptides predicted as antimicrobial and 200 peptides predicted as non-antimicrobial by AGRAMP. 180 out of 200 AGRAMP AMP predictions (90%) were predicted as AMP by AMPDiscover and 199 out of 200 NOAMPs (99.5%) were predicted as NOAMP by AMPDiscover. These results demonstrate that the AGRAMP predictions are generally in reasonable to good range of agreement with other AMP prediction methods, with the level of discrepancies usual for these diverse approaches.

**Table 4:** Comparison of antimicrobial peptide (AMP) predictions by different machine learning models.

	AMP^*^	Non-AMP (NOAMP)^**^
AGRAMP	10,000	10,000
AMP Scanner v.2.0	6,840	9,991
CAMP-RF	2,900	9,963
MACREL	4,541	9,975

^*^Machine learning models were employed to predict the top 10,000 AMPs predicted from AGRAMP. ^**^Machine learning models were employed to assess the top 10,000 non-AMPs predicted by AGRAMP.

### Growth inhibitory effect of predicted AMPs on *Spiroplasma citri*

**Figure 2:**
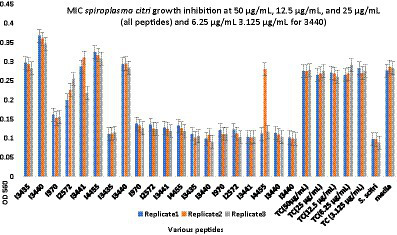
Minimum inhibitory concentration (MIC) assays of peptides predicted from the intergenic region of citrus genome for *Spiroplasma citri* Growth Inhibition. After 48-h incubation, MICs were tested with 3 replicates (Series1, Series2, Series3). The peptide concentration was at 50, 25, and 12.5 μg/mL. TC, Tetracycline; *S. citri*, *S. citri* only; media, LD8A3 media only.

**Figure 3:**
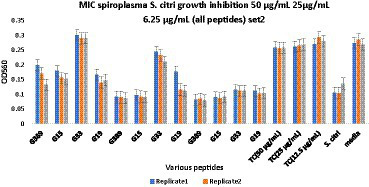
Minimum inhibitory concentration (MIC) assays of peptides derived from the whole citrus genome for *Spiroplasma citri* growth inhibition. The peptide concentration was 50, 25, and 6.25 μg/mL. After 48-h incubation, MICs were tested with 3 replicates (Series1, Series2, Series3). TC, Tetracycline; *S. citri*, *Spiroplasma citri* only; media, LD8A3 media only.

Minimum inhibitory concentration (MIC) testing is an essential *in vitro* assay used to determine the effectiveness of antimicrobial agents against specific microorganisms. In the present study, MIC assays were performed to evaluate the inhibitory properties of a small subset (Table 5) of predicted AMPs (10) against *S. citri* growth. These selected AMPs include those predicted from the intergenic regions (Set1, Figure 2) and the whole genome of citrus (Set2, Figure 3). Previous studies had established a correlation between spiroplasma culture acidity and OD_560_ values using phenol red as an indicator dye (Tully et al., 1977; Wei et al., 2022). The color change of the phenol red from pink to yellow indicates that the growth of *S. citri* was not inhibited. The negative controls (*S. citri* with medium and phenol red alone, without AMP) showed an OD_560_ range of 0.087–0.089 (yellow, Figures 2, 3; Supplementary Tables S2, S3). Conversely, in the presence of an active AMP or tetracycline (positive control), the growth of *S. citri* cells is inhibited, resulting in minimal or no change in culture acidity and the phenol red dye remaining red. For example, tetracycline, as the positive control, the inhibitory properties were observed with OD560 values ranging from 0.240 to 0.279 (Figures 2, 3; Supplementary Tables S2, S3).

**Table 5:** Putative antimicrobial peptides (AMPs) predicted from the citrus genome and selected for synthesis.

AMP ID		Peptide	Length (aa)
AMPs from the intergenic regions of citrus genome	I2572	MLKCHLVGFVRRLIN	15
	I3441	MLLQRLIFKPIRIIWHT	17
	I4455	MMKKVIKLQKMIALGKIVKRFSLY	24
	I970	MKSIKIKIKRLNSKNKKILILIFI	24
	I3435	MFLLRRILKKLRTIFIQ	17
	I3440	MILSVLKIFGVFRKRSRGN	19
AMPs from the whole citrus genome	G389	MGFLLKTLSHIRRVIRLII	22
	G15	MLNLKLIRLLRHRFAI	16
	G33	MIVRIAIRRFLKGKRQIVKI	16
	G19	MVSHLFCFKFIRNLRFKKIR	17

Compared with positive and negative controls, peptides I3435, I3440, I3441, I970, I4455, I2572, G15, G33, G19, and G389 significantly inhibited the growth of *S. citri* cells (Figures 2, 3). The peptides G33, I3435, I3440, I3441, and I4455 peptides showed particularly strong inhibition of *S. citri* growth (Supplementary Tables S2, S3). The MIC for all these peptides ranged from 12.5 μg/mL to 50 μg/mL at 48 h. The concentrations are given in μg/mL instead of μM because tetracycline, used as the reference control, is traditionally cited in μg/mL. Out of 20 synthesized predicted AMPs, only 10 exhibited strong inhibitory activity against *S. citri*. The remaining peptides either demonstrated resistance or weak inhibition against *S. citri* growth (data not shown). It is worth noting that the other 10 peptides that did not exhibit strong resistance to *S. citri* may still possess inhibitory activity against other bacteria.

### Machine learning—what features are important in the random forest algorithm models?

Several key features were identified as important in predicting AMPs by the Random Forest Algorithm (Table 6). These important features include hydrophobic residues (L, highlighted in yellow), in 3-gram 9-letter, and positively charged features in both the 3-gram charge (B, highlighted in red). It is interesting to note that the 9-letter alphabet used in this study, with mappings such as ED ≥ E, QTSNH ≥ Q, LMIVAF ≥ L, G ≥ G, W ≥ W, C ≥ C, RK ≥ R, Y ≥ Y, and P ≥ P, resulted in overrepresentation of hydrophobic residues (L) and certain amino acids with large groupings like glutamine (Q) and glycine (G). These findings align with the understanding that hydrophobic amino acids play a crucial role in the antimicrobial activity of AMPs, as discussed in the introduction. In the 3-gram 9-letter model, which comprised 729 features, the top 22 features were identified and analyzed. It is not surprising that hydrophobic residues (L) and glutamine (Q) were overrepresented, considering their abundance and importance in AMPs. Interestingly, the analysis did not reveal a high occurrence of clustered charged residues like RRR or RRQ in the 3-gram 9-letter alphabet. This suggests that other combinations of features, particularly hydrophobic and charged residues, are more influential in the prediction of AMPs. The antimicrobial APD database exhibits high frequencies of amino acids L (8.26), G (11.51), and K (9.51), which are commonly associated with alpha helices (Wang et al., 2022). Therefore, it might be expected that these residues would appear as top hits predicted by N-grams.

**Table 6:** Features of high importance in the Random Forest models: 3-gram 9-letter, and 3-gram charge.

No.	3-gram 9-letter	Importance	3-gram charge	Importance
1	LLG	2.62E-02	ZJJ	0.0996
2	ELQ	2.36E-02	JZJ	0.0877
3	LGR	2.34E-02	JJZ	0.0872
4	GLL	2.28E-02	JJJ	0.0832
5	LLL	2.14E-02	JJB	0.0824
6	EEL	1.74E-02	BJJ	0.081
7	QLQ	1.68E-02	JBJ	0.0705
8	QLE	1.62E-02	ZZJ	0.0562
9	QEL	1.60E-02	JBB	0.0385
10	LLE	1.47E-02	ZJB	0.0367

L represents hydrophobic residues; R represents positively charged residues in 3-gram 9-letter; B represents the positively charged features in 3-gramCharge. Please see the Materials and methods for the alphabet.

### Aggregation propensity and effectiveness of predicted AMPs

Furthermore, the relationship between aggregation propensity and antimicrobial peptide (AMP) activity was investigated. The Normalized a4v Sequence Sum for 100 residues (Na4vSS) was employed as a measure of *in vivo* aggregation propensity. Two categories of AMPs including positive (effective AMPs) and negative (ineffective AMPs) were used. The positive AMPs include AMPs reported as effective against *Spiroplasmas* in the literature (Béven et al., 1997, 2003; Wei et al., 2022; A, Table 7), and AMPs that were effective in the laboratory assay conducted in the current study (B, Table 7). The negative (Ineffective) AMPs encompass (i) Predicted AMPs from the N-gram program (AGRAMP) and other published AMP prediction programs (AMPScanner or MACREL or CAMP-RF) that were found to be ineffective in laboratory assays (C, Table 7); (ii) Ineffective peptides from previous studies (D, Table 7); and (iii) a peptide predicted to be ineffective by the N-gram program in the current study (E, Table 7). The present study compared positive data (effective AMPs) with negative data which often goes unpublished (Wang et al., 2022). Notably, a strong pattern formed, indicating that the predicted AMPs that were effective in the laboratory assay exhibited a positive aggregation propensity score.

**Table 7:** Aggregation values of AMPs, predicted AMPs, and predicted NOAMPs.

Peptide name	Normalized a4v sequence sum for 100 residues (Na4vSS):	MIC	Lab assay
LK12_3.6 (Bevin2002)	54.3	25 μm	A
LK15_3.6 (Bevin2002)	52.5	6.25 μm	A
LK15_W14_3.6 (Bevin2002)	50.7	6.25 μm	A
LK_Scrambled (Bevin2002)	50.7	100 μm	A
LK9_3.6 (Bevin2002)	46.9	100 μm	A
I3441	44.9	25 ug/mL	B
I3435	43.5	50 ug/mL	B
G389	42	50 μg/mL	B
LK16_W15_3 (Bevin2002)	41.7	6.25 μm	A
Caerin_11_APD0240 (Wei2021)	37.6	50 × 2^−7^ μg/mL	A
I4455	30.5	12.5 ug/mL	B
I4992	30.2	Resistant	C
I2572	29.6	25 ug/mL	B
I970	28.8	25 ug/mL	B
G19	26.8	50 ug/mL	B
G15	23.6	50 ug/mL	B
G33	22.7	25 ug/mL	B
I3440	21.9	25 ug/mL	B
Mellitin (Bevin1997)	16	0.39-.78 μm	A
I3775	13.6	Resistant	C
P3 (Bevin1997)	5.7	100 μm	A
Novispirin_T7_APD2710 (Wei2021)	4.8	50 × 2^−7^ μg/mL	A
P1 (Bevin1997)	1.8	100 μm	A
P2 (Bevin1997)	−1.3	100 μm	A
Tricholongin_APD2866 (Wei2021)	−1.9	Resistant	D
JM133 (Bevin1997)	−2.9	Resistant	D
G10159	−4	Resistant	C
G196	−4.8	Resistant	C
JM123 (Bevin1997)	−5	Resistant	D
I5196	−5	Resistant	C
G221	−13.3	Resistant	C
G66	−20.4	Resistant	C
I2769	−22.7	Resistant	C
G9994	−23.8	Resistant	C
G54	−31.2	Resistant	E

**Figure 4:**
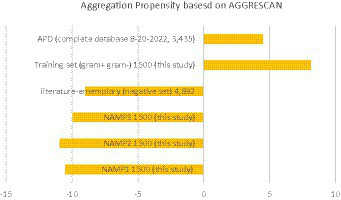
Aggregation summary using AGGRESCAN (Conchillo-Solé et al., 2007; Torrent et al., 2011; de Groot et al., 2012). Average aggregation values per 100 residues using the AGGRESCAN program on the entire APD database, the training set from this study, and the negative control sets as a comparison. NAMP (non-AMP)—peptides that are not predicted as AMPs.

Additionally, this study evaluated the aggregation propensity of the entire ADP database and compared it to negative datasets from this study and two negative datasets from published literature (Veltri et al., 2018; Sidorczuk et al., 2022). Although the pattern was not as pronounced and showed a higher standard deviation (Figure 4), AMPs tended to have a positive aggregation score, while negative data tended to have a negative aggregation score. These findings suggest that there may be a correlation between aggregation propensity and AMP effectiveness. The comparison of aggregation propensity between positive and negative datasets supports this observation, although with some variability. It highlights the potential significance of considering aggregation propensity in the design and assessment of AMPs for antimicrobial applications.

### AGRAMP—web interface

A web-based program called AGRAMP (see text footnote 1) was developed using PHP, leveraging the models created in this study. The AGRAMP was designed to analyze short amino acid sequence in FASTA format. Users can input their sequences into a text box and select the desired parameters from pull-down menus, including options for 2-gram and 3-gram models, as well as 9-letter and 3-letter alphabets employed in this study. The program generates an output table that presents the submitted peptide with a confidence prediction of the probability of that peptide as a possible AMP. This online AGRAMP tool will allow users to assess their unknown peptide being AMPs using N-gram analysis. The training and validation sets used in this paper are available on the server.

## Discussion

Humanity’s food supply faces continuous challenges from bacterial pathogens that not only threaten crop yields but also diminish the quality of agricultural commodities. Implementing control measures against these pathogens often leads to significant increases in production costs. While antibiotics can effectively suppress plant pathogens, their use on a large scale in agricultural production is impractical due to their prohibitive cost and the risk of microbial resistance in the long run. AMPs have garnered significant attention as promising alternatives to traditional antibiotics for combatting plant pathogenic bacteria in agriculture and the environment. Their unique properties, such as broad-spectrum activity, rapid killing kinetics, and low propensity for developing resistance, make them attractive candidates for developing novel strategies to manage plant diseases. However, the process of identifying potent AMPs through traditional laboratory assays is often time-consuming, labor-intensive, and costly. To address these challenges, the present study proposes a bioinformatics approach that leverages machine learning models based on the N-gram method to predict and select AMPs with antimicrobial activity against plant pathogens.

In this study, N-gram models, specifically 2-gram and 3-gram, were employed to capture fundamental sequence patterns inherent in antimicrobial peptide. Furthermore, the impact of reduced alphabets, consisting of either a 9-letter or a 3-letter representation was also examined. These choices are made to optimize the performance of the machine learning models in accurately predicting AMPs. The performance of the proposed model is rigorously evaluated through cross-validation and the Mathew’s correlation coefficient (MCC), ensuring its reliability and predictive power. The results demonstrate the effectiveness of the machine learning model in accurately predicting AMPs and effectively distinguishing between AMPs and non-AMPs (NOAMPs; Tables 1, 2). The 3-gram 9-letter model slightly outperformed other models, achieving a high cross-validation score 0.91, indicating accurate classification of AMPs. The corresponding MCC value 0.79 further reinforces the model’s robustness in accurately discriminating between AMPs and NOAMPs. Additionally, the 3-gram 3-letter, 2-gram 9-letter, and 2-gram 3-letter models exhibit satisfactory performance in AMP classification (Tables 1, 2).

Building upon the success of the machine learning models, they were employed to predict putative AMPs encoded by intergenic regions and small ORFs within the citrus genome. By exploring these uncharacterized regions, the study taps into the vast potential of the citrus genome to provide novel AMP candidates. These predicted AMPs are then subjected to experimental validation against *S. citri*, the causative agent of citrus stubborn disease. The experimental results confirm the antimicrobial activity of the selected AMPs against the target bacterium, further bolstering the predictive capability of the machine learning models (Figures 2, 3).

**Figure 5:**
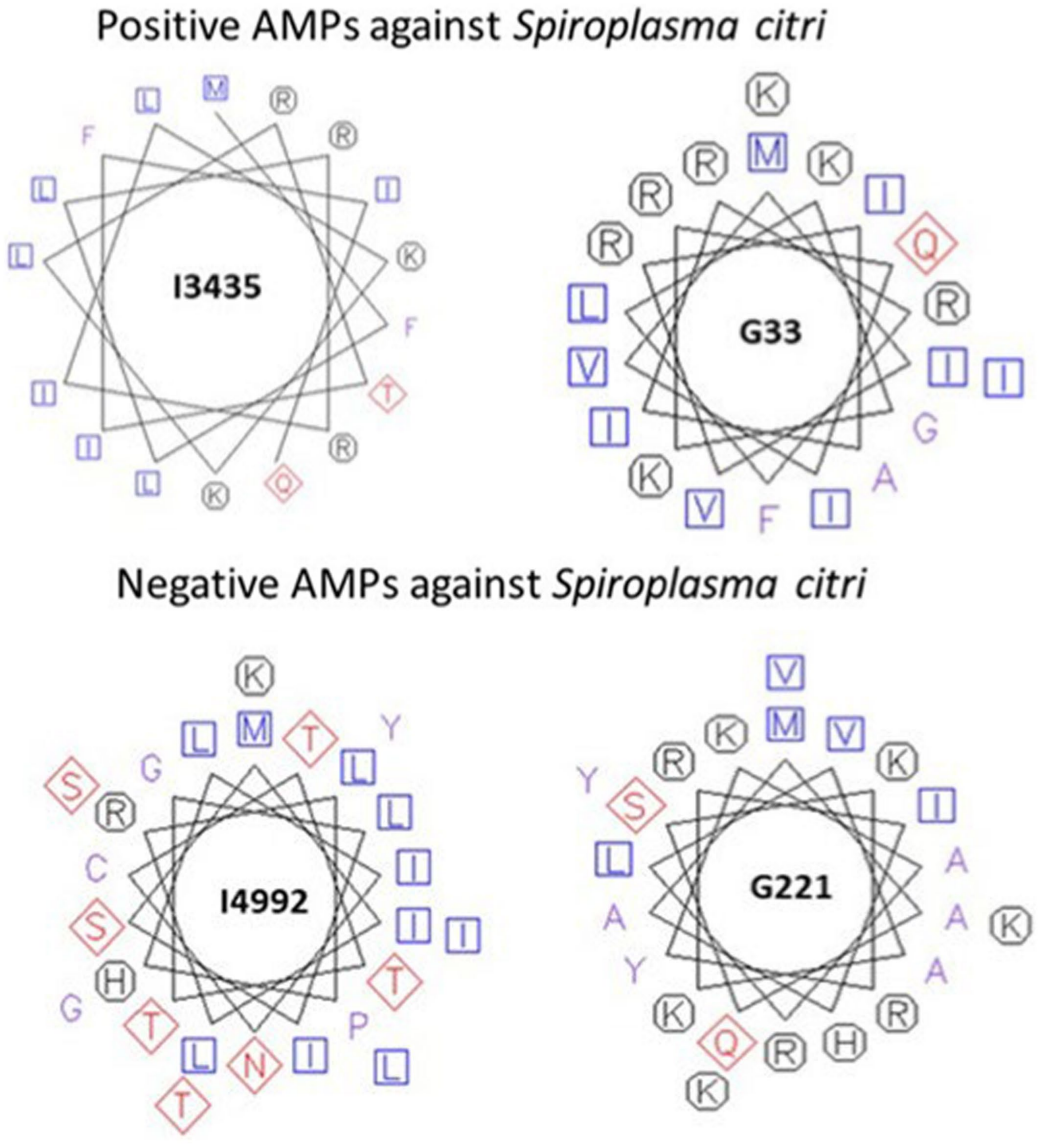
Pepwheels of predicted antimicrobial peptides (AMPs) tested in minimum inhibitory concentration (MIC) assays. Top panel represents pepwheels of selected AMPs with high activity in MIC assay. Bottom panel represents pepwheels of selected AMPs with no activity in MIC assay. Blue squares represent non-polar amino acids. Red Diamonds represent polar amino acids and Black octagons represent charged amino acids.

**Figure 6:**
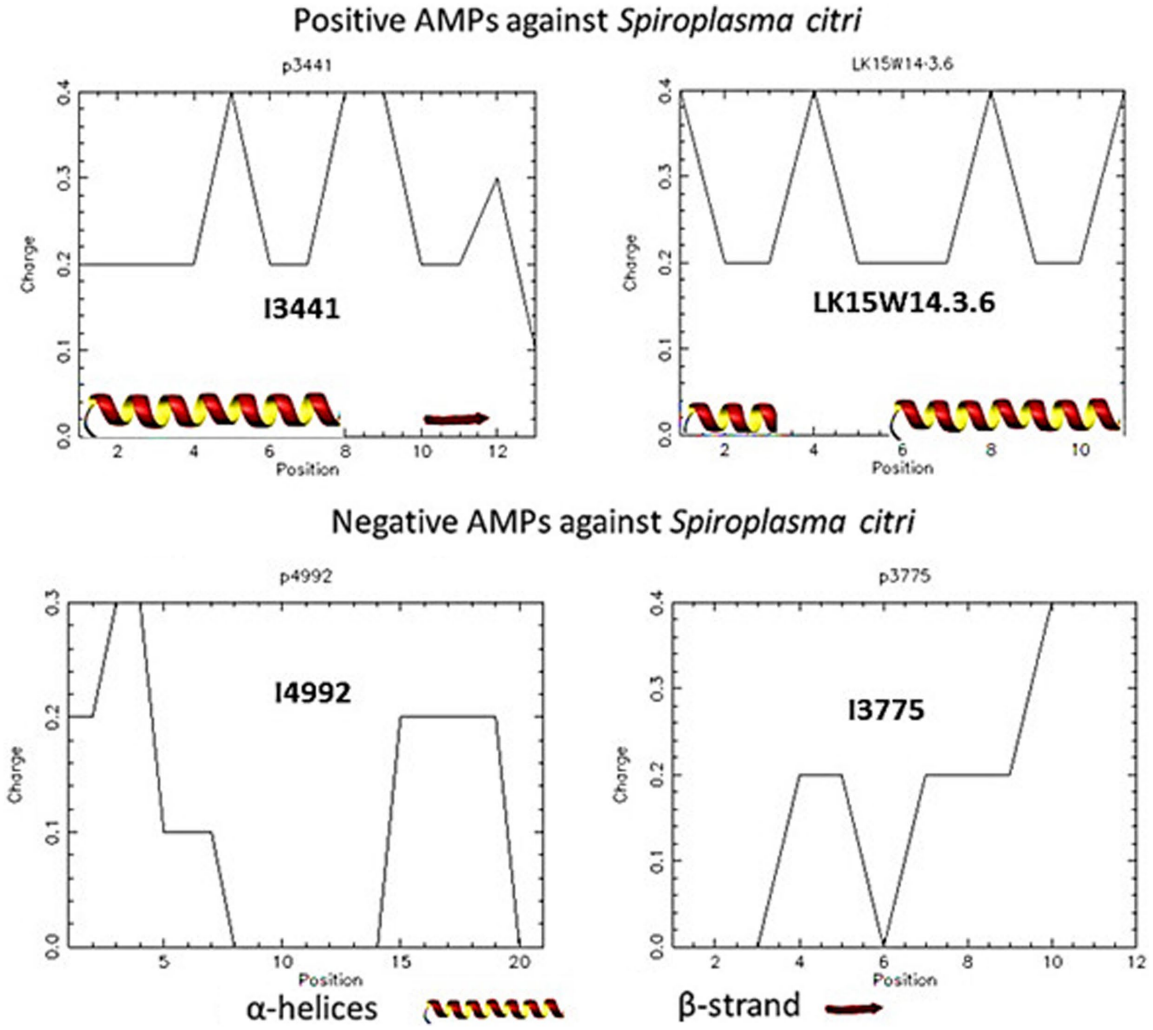
Charge density analysis of predicted antimicrobial peptides (AMPs) tested in minimum inhibitory concentration (MIC) assays. The top panel displays the charge density of AMPs with positive activity against *Spiroplasma citri*. The bottom panel demonstrates the charge density of AMPs with negative activity against *S. citri*. The top right panel shows the charge density of AMP affecting *Spiroplasma citri* documented in the previous literature. The secondary structure of each AMP is indicated at the bottom of the respective graph.

The properties of the peptides that showed inhibition against *S. citri* were explored through pepwheels, charge density graphs and examination of the secondary structure. The pepwheels of the peptides I3435 and G33 show that charged and hydrophobic residues have an alternating pattern compared to I4992 and G221 which show less of a discernable pattern of shapes (Figure 5). Though the AMP pepwheels do not present a unified pattern, there are patterns that are more common in the positive set than the negative set such as the alternating charged and hydrophobic residues. Similarly, the charge density graphs of the AMPs that did work in our laboratory *S. citri* growth inhibition assay have a series of peaks and valleys (Figure 6). The pattern of peaks and valleys in the charge density graphs also appear to be very similar to the charge density graphs of AMPs that are effective against *S. citri* in the literature as shown by LK15W14.3.6 (Béven et al., 2003). Such results suggest that taking the position information and the charge information and generating features from the charge density plot and pepwheels would further aid in AMP prediction. Further, it is known that the secondary structure is important in AMP activity. AMPs successful in *S. citri* assay generally had secondary structures such as alpha helices (Table 8).

**Table 8:** Secondary structure prediction of predicted AMPs using JPred4 tool.

Predicted AMPs using JPred4	
I2572	MLKCHLVGFVRRLIN ||||||||||||||| ---------HHHHEE
I3440	MILSVLKIFGVFRKRSRGN ||||||||||||||||||| HHHHHHHHHHHHH------
I3441	MLLQRLIFKPIRIIWHT ||||||||||||||||| HHHHHH-----EEEE-H
I3435	MFLLRRILKKLRTIFIQ ||||||||||||||||| HHHHHHHHHHHHHHHHH
I4455	MMKKVIKLQKMIALGKIVKRFSLY |||||||||||||||||||||||| E---------EEEE--EE---EEE
G15	MLNLKLIRLLRHRFAI |||||||||||||||| HHHHHHHHH—HHHHHH
G33	MIVRIAIRRFLKGKRQIVKI |||||||||||||||||||| HHHHHHHHHHH--HHHHHHH
G389	MGFLLKTLSHIRRVIRLII ||||||||||||||||||| HHHHH---------HEEHH
G19	MVSHLFCFKFIRNLRFKKIR |||||||||||||||||||| ---EEEH-------------

Peptides found in literature	
P1 (Béven et al., 1997)	MGLGLHLLVLAAALQGAWSQPKKKRKV ||||||||||||||||||||||||||| ------HHHHHHHHH------------
P2 (Béven et al., 1997)	MGLGLHLLLAAALQGAKKKRKV |||||||||||||||||||||| ----------------------
P3 (Béven et al., 1997)	MGLGLHLLVLAAALQGAKKKRKV ||||||||||||||||||||||| -----HHHHHH------------
LK15_3.6 (Béven et al., 2003)	KLLKLLLKLLLKLLK ||||||||||||||| HHHHHH--HHHHHHH
LK15W14_3.6 (Béven et al., 2003)	KLLKLLLKLLLKLWK ||||||||||||||| HH----HHHHHHHHH

Furthermore, the present study revealed an intriguing relationship between protein aggregation and AMPs (Figure 4; Table 7). Torrent et al. (2011) conducted an interesting analysis to calculate peptide aggregation in AMPs using AGGRESCAN software, which effectively predicted aggregation in bacteria. This algorithm utilizes an amino acid aggregation-propensity scale and is based on the assumption that short sequence stretches modulate protein aggregation, resulting in hotspots of aggregation (Conchillo-Solé et al., 2007; de Groot et al., 2012). These facts indicated that AMPs might reduce their aggregation in a solution but promote aggregation in a more hydrophobic environment, such as the bacterial cell membrane (Torrent et al., 2011). However, it is puzzling why some peptides predicted to be effective *in silico* failed to demonstrate activity *in vitro* laboratory assays against live bacterial cells. While the lack of a secondary structure was initially considered as a potential explanation, this explanation did not hold true for all cases. An alternative hypothesis was proposed, suggesting that peptides with positive predictions for AMP activity might have failed *in vitro* due to a low aggregation propensity score. This could result in their inability to aggregate effectively and form the requisite pore structure responsible for depolarizing the cell membrane. In contrast, peptides demonstrating efficacy in laboratory assays exhibited higher aggregation scores. Additionally, for peptides with low aggregation scores that still exhibited activity, it was postulated that alternative mechanisms might be employed to inhibit cell growth, such as targeting cytoplasmic components, independent of extensive aggregation for functionality. Moreover, AMPs have the capacity to target multiple cellular components, including bacterial cell walls and ribosomes, further contributing to their antimicrobial activity (Wang et al., 2022).

Using *Spiroplasma citri* as an example, the studies conducted by Béven et al. (1997, 2003) revealed that three peptides (P1, P2, P3) inhibited *S. citri* at a MIC concentration of 100 μM. In contrast, the 2003 study found that many of the LK peptides (LK12_3.6, LK15_3.6, LK15_W14_3.6, LK_Scrambled, LK9_3.6) had a MIC concentration of 6.25 μM. Intriguingly, the LK peptides had higher aggregation propensities (in the high 40s and 50s), while the peptides in the 1997 study had slightly positive (5.7, 1.3) and negative (−1.3) aggregation scores when input into AGGRESCAN. The positive control, Melittin, had a positive aggregation score of 16. Similarly, in a study by Wei et al. (2022), peptides that tended to work (Caerin_11_APD0240) exhibited positive aggregation, whereas those that did not (Tricholongin_APD2866) had zero or negative aggregation values. Although not statistically significant, these findings, combined with other studies, suggest a correlation between aggregation propensity and AMP effectiveness in laboratory assays. This observation warrants further investigation, as it could provide valuable insights into the mechanisms and targets of antimicrobial peptides.

In the field of machine learning, reported high accuracies often do not align with the outcomes of real-world empirical testing (Wang et al., 2022). One reason for this discrepancy is that most AMP prediction programs are trained on general AMP data in their training sets, and their outputs typically classify peptides as either AMP or non-AMP without providing further details on the activity or effectiveness of the predicted AMPs. To enhance the accuracy of AMP prediction *in vivo* studies, the future of AMP prediction in machine learning must involve the integration of laboratory data into the algorithms. Protein aggregation, as discussed earlier, plays a significant role in AMP activity. A study investigating antibiotic design strategies in *Staphylococcus aureus* found that bacterial peptides aggregate when they enter and accumulate in the bacterial cytosol, and the study also explored the hemolytic effects of the peptides (Bednarska et al., 2016). Machine learning could be effectively applied to similar studies exploring the mechanisms and aggregation of host-cell cytotoxicity and hemolysis. For instance, N-gram features of peptides implicated in pore formation could be compared with those implicated in targeting cytoplasmic targets. Additionally, N-grams of peptides known to have hemolytic activity safe for mammalian cells could be contrasted with those that exhibit hemolytic activity harmful to mammalian cells. By incorporating such biological phenomena and their associated N-gram features into the model, machine learning can better predict AMP activities and functions.

One potential reason why existing AMP prediction programs do not incorporate these aspects is the lack of standardization in reporting laboratory results (Wang et al., 2022), along with the limited availability of large, standardized training sets. Moreover, many computational AMP studies often conclude with AMP/NOAMP predictions and statistical analyses, without delving into the underlying mechanisms or exploring beyond MIC values and bacterial inhibition. Thus, the next frontier in AMP prediction involves exploring how past laboratory experiments can be effectively harnessed in machine learning or designing large-scale future laboratory experiments to facilitate the machine learning process. To design an optimal peptide, integration with other existing programs may also be necessary. By incorporating more comprehensive and standardized laboratory data, machine learning can pave the way for more accurate AMP prediction and contribute significantly to the design and discovery of novel antimicrobial peptides with enhanced effectiveness and specificity.

To aid in widespread accessibility and usability, we developed a publicly available online resource named AGRAMP (Agricultural N-grams Antimicrobial Peptides). AGRAMP enables users to input peptide sequences and obtain predictions of putative AMPs. This resource serves as a valuable tool for researchers and practitioners in the field, offering a convenient and efficient means of identifying and selecting potential AMPs. By democratizing access to the bioinformatics approach and machine learning models, AGRAMP accelerates the process of screening and selecting effective AMP candidates, thus contributing to the advancement of plant disease management in agriculture and the environment.

## Data availability statement

The datasets presented in this study can be found in online repositories. The names of the repository/repositories and accession number(s) can be found in the article/Supplementary material.

## Author contributions

JS: Data curation, Formal analysis, Investigation, Methodology, Software, Validation, Visualization, Writing – original draft. YZ: Formal analysis, Investigation, Methodology, Resources, Writing – review & editing. WW: Formal analysis, Investigation, Methodology, Resources, Writing – review & editing. IV: Conceptualization, Formal analysis, Investigation, Methodology, Project administration, Supervision, Visualization, Writing – review & editing.

## Footnotes

^1^http://omics.gmu.edu/agramp

^2^https://www.uniprot.org/

^3^http://www.camp.bicnirrh.res.in/

^4^http://aps.unmc.edu/AP/main.html

^5^https://aps.unmc.edu/

^6^http://weizhongli-lab.org/cd-hit/

^7^https://www.citrusgenomedb.org/bio_data/91

^8^https://bedtools.readthedocs.io/en/latest/

^9^https://www.ebi.ac.uk/Tools/st/emboss_transeq/

^10^https://www.compbio.dundee.ac.uk/jpred/

^11^https://www.bioinformatics.nl/cgi-bin/emboss/charge

^12^https://www.bioinformatics.nl/cgi-bin/emboss/pepwheel

^13^https://www.r-project.org/

^13^https://biocom-ampdiscover.cicese.mx/
