# Supplementary material for: AGRAMP: machine learning models for predicting antimicrobial peptides against phytopathogenic bacteria
Source: Front Microbiol. 2024 Mar 7;15:1304044. doi: 10.3389/fmicb.2024.1304044 (PMC10955071; doi:10.3389/fmicb.2024.1304044)
Supplement: Supplementary file 1 [file Table_1.DOCX]

S1. Putative peptides selected for synthesis based on color codes

AMP ID (predicted peptide)

| **AMP**  **ID** | **Peptide** | **len** | **AMP**  **ID** | **Peptide** | **len** |
| --- | --- | --- | --- | --- | --- |
| I970 | MKSIKIKIKRLNSKNKKILILIFI | 24 | I2769 | MSRKLRKGKRQLKSPRFGKIVI | 22 |
| I2572 | MLKCHLVGFVRRLIN | 15 | I3775 | MIDNKIGLVVKWLRSK | 16 |
| I3435 | MFLLRRILKKLRTIFIQ | 17 | I4992 | MILRLPHLINCTTTGLISKITSYLG | 25 |
| I3440 | MILSVLKIFGVFRKRSRGN | 19 | I5196 | MERRRLAAKLANKLVRIGWIG | 21 |
| I3441 | MLLQRLIFKPIRIIWHT | 17 | G54 | MLIMRKIKKKKRKNRHI | 17 |
| I4455 | MMKKVIKLQKMIALGKIVKRFSLY | 24 | G66 | MARKAISKVIVGTRRKKKK | 19 |
| G15 | MLNLKLIRLLRHRFAI | 16 | G196 | MITGRQKALKFIKKKVGGIQ | 20 |
| G19 | MVSHLFCFKFIRNLRFKKIR | 20 | G221 | MAQSKRYKARLVAKRIHAVKKY | 22 |
| G33 | MIVRIAIRRFLKGKRQIVKI | 20 | G9994 | MCRRKGGLIGRAPPCRGRLAC | 21 |
| G389 | MGFLLKTLSHIRRVIRLII | 19 | G10159 | MILKRVFRMKNKKASV | 16 |

S2. Laboratory assay summary and MIC of predicted antimicrobial peptides tested for *S. citri* inhibition (Set1).

| AMP_ID (predicted) | SpiroC  OD_560_  (Avg) | SpiroC  p-value | SpiroC  MIC  (48hrs) | SpiroC  p-value |  |
| --- | --- | --- | --- | --- | --- |
| LD8A3 (media only) | 0.258 | - | - | - |  |
| Tetracycline | 0.24 | - | - | - |  |
| *S. citri*-NOAMP | 0.087 | - | - | - |  |
| I3435 | 0.245 | 5.60E-11 | 50 µg/mL | 1.80E-15 |  |
| I3440 | 0.225 | 4.20E-10 | 25 µg/mL | 1.80E-15 |  |
| I970 | 0.231 | 2.30E-10 | 25 µg/mL | 0.001 |  |
| I2572 | 0.249 | 4.50E-11 | 25 µg/mL | 0.003 |  |
| I3441 | 0.141 | 0.087 | 25 µg/mL | 0.001 |  |
| I4455 | 0.261 | 2.40E-11 | 12.5 µg/mL | 4.70E-06 |  |

S3. Laboratory assay summary and MIC of predicted antimicrobial peptide on *S. citri* cell growth inhibition (Set2)

| AMP_ID (predicted) | OD_560_  (Avg) | p-value | MIC  (48hrs)  Resistant (R) | p-value |  |
| --- | --- | --- | --- | --- | --- |
| LD8A3 (media only) | 0.288 | - | - | - |  |
| *S. citri*-NOAMP | 0.089 | - | - | - |  |
| Tetracycline | 0.279 | - | - | - |  |
| G389 | 0.157 | 6.40E-11 | 50 µg/mL | 9.40E-05 |  |
| G15 | 0.228 | 2.00E-16 | 50 µg/mL | 0.00016 |  |
| G33 | 0.274 | 2.00E-16 | 25 µg/mL | 1.10E-09 |  |
| G19 | 0.126 | 5.20E-08 | 50 µg/mL | 0.002 |  |
